# A Network Pharmacology-Based Approach to Investigate the Novel TCM Formula against Huntington's Disease and Validated by Support Vector Machine Model

**DOI:** 10.1155/2018/6020197

**Published:** 2018-12-11

**Authors:** Wenjie Dai, Hsin-Yi Chen, Calvin Yu-Chian Chen

**Affiliations:** ^1^School of Intelligent Systems Engineering, Sun Yat-sen University, Shenzhen 510275, China; ^2^Department of Medical Research, China Medical University Hospital, Taichung 40447, Taiwan; ^3^Department of Bioinformatics and Medical Engineering, Asia University, Taichung 41354, Taiwan

## Abstract

Several pathways are crucial in Huntington's disease (HD). Based on the concept of multitargets, network pharmacology-based analysis was employed to find out related proteins in disease network. The network target method aims to find out related mechanism of efficacy substances in rational design way. Traditional Chinese medicine prescriptions would be used for research and development against HD. Virtual screening was performed to obtain drug molecules with high binding capacity from traditional Chinese medicine (TCM) database@Taiwan. Quantitative structure-activity relationship (QSAR) models were conducted by MLR, SVM, CoMFA, and CoMSIA, constructed to predict the bioactivities of candidates. The compounds with high-dock score were further analyzed compared with control. Traditional Chinese medicine reported in the literature could be the training set provided for constructing novel formula by SVM model. We tried to find a novel formula that can bind well with these targets at the same time, which indicates our design could be highly related to the HD. Additionally, the candidates would validate by a long-term molecular dynamics (MD) simulation, 5 microseconds. Thus, we suggested the herbs* Brucea javanica, Holarrhena antidysenterica*,* Dichroa febrifuga, Erythrophleum guineense, *etc. which contained active compounds might be a novel medicine formula toward Huntington's disease.

## 1. Introduction

Huntington disease (HD) is a kind of involuntary movement, mental disorders, and progressive dementia as the main clinical features of dominant hereditary neurological degeneration disease. The pathology is identified as the protein named Huntingtin [1], which is produced by the site 63 of fourth chromosome. Pathological changes are characterized by the loss of nerve cells in the striatum and cerebral cortex. The novel research points out that the link between peripheral biology and neurodegeneration are shown in other chronic neurodegenerative diseases, suggesting that the modulation of peripheral targets may provide novel ways of therapeutic development [2]. The information here also indicated a multitargets way against the disease.

Neurodegenerative diseases were studied together frequently [3] because of the common features such as misfolding [4]. Studies had constructed a single cell model that greatly promotes the development of neurodegenerative diseases [5]. Many genes are involved in HD [6]. Correspondingly, the treatment of HD is related to multiple ideas, such as antiapoptotic effect [7], antioxidant [8, 9], improving memory by enhancing hippocampal synaptic plasticity [10], and immunotherapies [11].

A novel method of merging traditional Chinese medicine (TCM) with network pharmacology-based could be a reliable way to overcome disease. In the past decades, the single-target drugs sometimes did not achieve favorable therapeutic effects. It would prefer to use the network pharmacology-based approach to develop a treatment in a multitargeted, multidrug manner [12]. Recently, there is a concept about treating the whole body in HD [2]; brain cells are under attack, causing a stress response in cells far from the brain [13].

Most diseases are associated with multiple target proteins, and it is difficult to achieve proper therapeutic results with a single target. Similar to evidence-based medicine, protein-protein interaction (PPI) network is in line with both western medicine and traditional Chinese medicine. The theory of traditional Chinese medicine holds that the body is an organic whole, and its components are inseparable in structure. This association includes the PPI in modern medicine, which is interdependent in physiological conditions and influences each other in pathology [14].

TCM is the quintessence of China. After thousands years of accumulation, there are enough clinical examples to confirm its rationality [15]. Therefore, the return to traditional Chinese medicine is imperative. Developing a novel type of traditional Chinese medicine prescription could guide the treatment of diseases. Combined with the modern biotechnology computer simulation technology, it could filter out the leading small molecules from the TCM database within antidisease activity. As traditional Chinese medicine has the advantages of relative low toxicity [16], we propose to return to traditional Chinese medicine to screen out the appropriate formula to treat some diseases and even critical diseases. TCM had precedents for the treatment of diseases [17], even some of which modern medicine cannot solve.

The energy metabolism of medium-sized thorny neurons in the striatum of HD patients is affected by the abnormal energy metabolism that rapidly dissipates neurons and is susceptible to excite-toxicity and ultimately damage the cortical neurons, so abnormal energy metabolism may also be related to the pathogenesis of HD [18]. Knockdown of the mitochondrial chaperone mtHSP70 causes a unique fat-mediated stress response that corrects the cellular protein folding. Drugs that activate this mitochondrial-to-cytosolic stress response could play a protective role [13, 19]. In another word, mtHSP70 inhibitors could reduce protein misfolding, which is a key pathogenesis in lots of neurological diseases.

Another idea to cure HD is to protect neurons. The neuronal stress-protective transcription factor HSF1 will be abnormal degradation [20], which replaces the damage to protection factors. Therefore, we should protect the heat shock transcription factor 1 (HSF1) through a way of cutting the hurt of HSF1. Casein kinase II subunit alpha' (CK2 alpha', CSNK2A2, CK2A2) inhibitors play a pivoted role to protect HSF1 [20].

**Figure 1:**
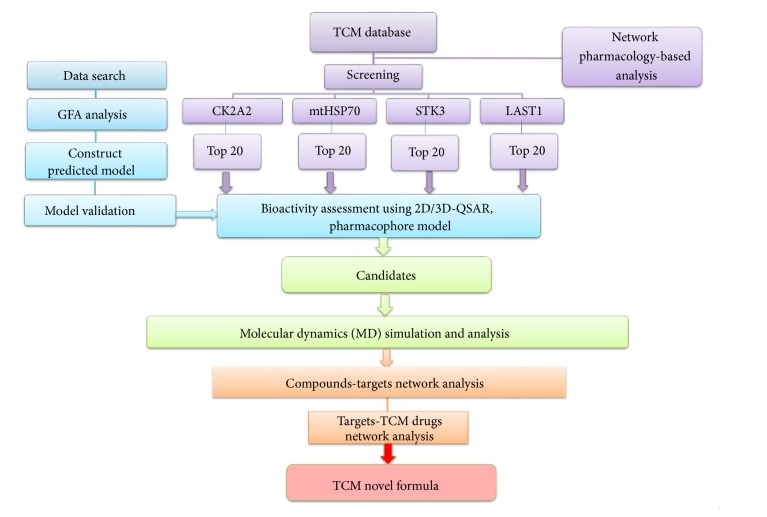
Flowchart of experiment process.

The biological significance of YAP binding to TEAD has been more and more clear, but the three-dimensional structure of both and the molecular mechanism of their action remain unclear. In recent year, this pathway is regarded as a potential target toward HD [21]. Huntington's disease (HD) gene product Huntingtin (Htt) selectively induces new forms of necrotic cell death, in which the endoplasmic reticulum (ER) expands and the cell body asymmetric balloon is ultimately cracked. The special necrotic cell death is mediated by functional deficits in TEAD/YAP-dependent transcription instead of RIP1/3 pathway-dependent necroptosis [22]. The special necroptosis way depends on the way of transferring the YAP from TEAD to p73 through YAP phosphorylation [23]. It could be a therapeutic target against HD. The experimental protocol was aimed at developing a novel anti-HD TCM formula (Figure 1).

## 2. Materials and Methods

### 2.1. Network Pharmacology-Based Analysis

Based on the preliminary work, Kyoto Encyclopedia of Genes and Genomes (KEGG) database was utilized to generate the related proteins to construct a protein-protein interaction network. The hub protein should be identified in the pathway network. It was clear that the inhibition of Mitochondrial 70 kDa heat shock protein (mtHSP70, HAPA9, mortalin) and CK2A2 benefitted from HD resistance. Based on the multitarget idea, the related targets would be identified after network pharmacology-based analysis about YAP1 pathway. The YAP level would decrease in HD brain model [22], which indicates the downshift of YAP could be one of pathogenic factors. And the signal pathway could provide information of up-/down-adjust which can guide us to find the vital protein in the pathway network. Another 2 protein targets, Serine/threonine-protein kinase 3 (STK3) and Serine/threonine-protein kinase LATS1, were studied further.

### 2.2. 3D Structure Modeling for LATS1 Protein

The protein LATS1 has no enough structure information for structure-based drug design. The origin structure of LATS1 in PDB (5BRK:B) was further modeled to obtain a complete structure [24]. The binding site is set as a natural structure, which indicates that it was credible. The structure was verified in Ramachandran plot [25] and Verify profile-3D.

### 2.3. Docking Study of Target Proteins

The sequence of human CK2alpha' protein (residues 1-350) was acquired from the Uniprot Knowledgebase (P19784). The 3D crystal structure of human CK2alpha' protein, 5M56 (residues 1-350) was obtained from Protein Database Bank (PDB) website [26]. 5M56 was the latest CK2alpha' prototype structural which has the complete 3D structure used for ligand-protein docking. All compounds from TCM Database@Taiwan, the world largest TCM database [27], were applied to dock with 5M56 by using Accelrys Discovery Studio (DS) software. LigandFit program in DS was performed to conduct molecular docking procedure. The ATP binding area was set as a binding sites referring to the ATP-competitive inhibitors, and one of the known inhibitor was set as a control in our study [28]. HARvard Molecular Mechanics (CHARMm) force field was performed to minimize energy. Affinity would be considered in Dock score, where the ligands with high score in docking would be the candidates to interact with target. Multiple poses would be calculated, 11 scoring patterns would be considered, and the consensus score would be set as a comprehensive consideration.

The same way was descripted above; another two targets screened small molecules in the TCM database as well. The information of HSPA9 (mtHSP70) protein was gained from UniprotKB (P38646). 3D structure of HSPA9 was acquired in PDB (4KBO, 52-431). The binding pocket referred to the nucleotide-binding domain (NBD), which is an active binding region [29]. MST2 (STK3) protein in the PDB (4LGD, 4-491) was selected for studying similarly. A peptide could inhibit the homodimerization of MST2, which could further prevent the activity of MST2 by transautophosphorylation [30]. And the binding site could be referred from it. The LATS1 was docking employing our modeling structure mentioned above.

The screen result of four targets would be analyzed in a network to discover which compounds have effects on multiple targets.

### 2.4. Quantitative Structure–Activity Relationship (QSAR) Models and Predictive Work

The bioactivity was predicted through QSAR model [31]. The Genetic Function Approximation (GFA) algorithm [32], a method of searching for the optimal solution by simulating the natural evolutionary process, was utilized to look for the proper molecular descriptors and then used Calculate Molecular Property protocol to acquire relative properties in DS software. 2D-QSAR models were conducted through multiple linear regression (MLR) with Matlab and support vector machine (SVM) with libSVM. The way to evaluate these models was the value of square correlation coefficient (R^2^) which is calculated in the regression. Several molecules with known activity were used for external verification as well. The predictive models were chosen from the models which have high value of square correlation coefficient. And then the predictive models were performed to forecast each candidate drugs after docking.

The docking scores of candidates with CK2A2 were provided, as well as the docking score with other related proteins, mtHSP70, STK3, and LATS1. And the predicted activities or scores through several known compounds or traditional Chinese medicines about HD could also take a reference to the final drugs' selection. Some proteins (CK2A2 and STK3) also provided a control for comparing.

Thirty-one known ligands [33] for CK2 protein with IC50 information constructed the predicted model by SVM and MLR algorithm. Thirty-five ligands [34] for meHSP70 protein with IC50 information conducted the predicted model as well. The STK3 and LATS1 protein constructed the predicted models through the reported TCM formula, and the active ingredients would be gathered in our TCM database. The aim was to build prediction module through SVM and MLR algorithm to predict better medicines from the clinical medicines models.

### 2.5. 3D-QSAR Analysis

3D-QSAR was constructed by known ligands [33] for CK2 protein with Sybyl-X 1.1. These active ligands were superimposed. The Comparative Molecular Field Analysis (CoMFA) [35] and Comparative molecular similarity index analysis (CoMSIA) [36] models were constructed according to their activity and structural characteristics. The models were evaluated through cross-validation (CV). Residual between the measured and predicted values was calculated and the square sum of explained (SSE); F-test, coefficient of determination (R^2^), and q^2^ for cross-validation were referred as evaluation indexes of 3D-QSAR models. CoMFA analyzes the effects of stereoscopic fields and electric fields, while CoMSIA evaluates stereoscopic fields, electric fields, hydrophobic fields, and hydrogen bonds for acceptors and donors. Various field combinations had been studied separately and the best model would be further studied.

### 2.6. Pharmacophore Analysis and Cross Validation

Pharmacophore models with 38 compounds [33, 37, 38] were created by hypoGen protocol, which was to analyze spatial influence factors such as hydrogen bonds acceptors, hydrophobic interactions, and *π*-conjugated effect. The models were verified through leave-one-out cross validation (LOOCV) [39]. Fisher's randomization test was used to ensure that reliable hypotheses were built. To obtain a 95% confidence level, a total of 19 randomizations were required.

### 2.7. Molecular Dynamics Simulation (MDs)

In order to verify the results of docking screening, molecular dynamics simulations were performed on all candidate compounds screened above using gromacs5.0.4 program software with world's top computing resources, Tianhe No. 2 supercomputer for long enough (5000 ns) simulation. The 5000 ns is basically the longest simulation in the current stage (2018). The energy minimization was produced by the steepest descent algorithm with the maximum number of 5000 steps minimization. The NVT equilibration was set in 20 ns with each step of 2fs and constrained with Lincs algorithm. The NPT equilibration was running after NVT in total of 20 ns. All bonds (even heavy atom-H bonds) were constrained with Lincs algorithm at 300K. The long time molecular dynasty was set in 5000 ns (for LATS1 protein nearly 3500 ns because of the computing resources). With the analysis of root-mean-square deviation (RMSD), root mean square fluctuation (RMSF), mean square displacement (MSD), secondary structure of protein, solvent accessible surface area (SASA), radius of Gyration (gyrate), the residue contact map (mdmat), Hydrogen bond distance (H-bond), energy analysis, and torsion angle, even the cluster research, the candidates will be validated to acquire a reasonable conclusion. After all, please be aware of short time MD simulation. Several candidates were “flying away” from protein during 5000 ns, even though they interacted well with targets at the first period.

## 3. Results

### 3.1. Protein-Protein Interactions Network Analysis

From the interaction information about the known targets, some high-related targets could be further confirmed in the research. The decreased expression level of YAP in human HD brains neurons was known [22]. Up-adjust YAP level can be the treatment method.

**Figure 2:**
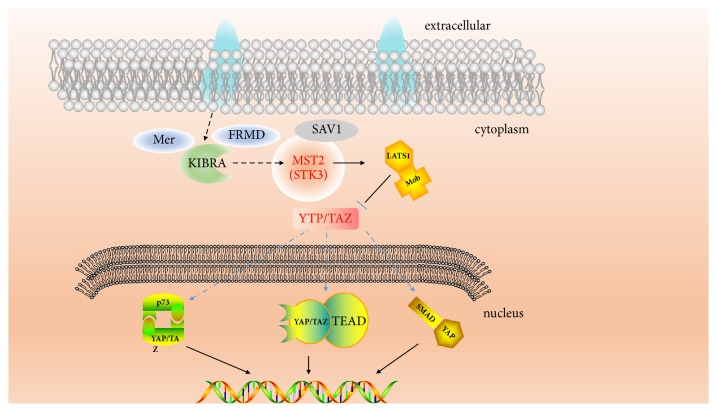
The pathway of YAP signal from KEGG pathway database.

The hub node in the pathway was important and probably got more interactions with other related proteins. The proteins in the pathway were gathered; it showed the related proteins which could adjust (Figure 2). It could be certain clearly which agonists or inhibitors could be designed from it. The key proteins could be found in the pathway. Here four proteins, CK2A2, mtHSP70, mst2 (STK3), and LATS1 were identified as the targets for the further research.

### 3.2. Modeling Structure Verification

**Figure 3:**
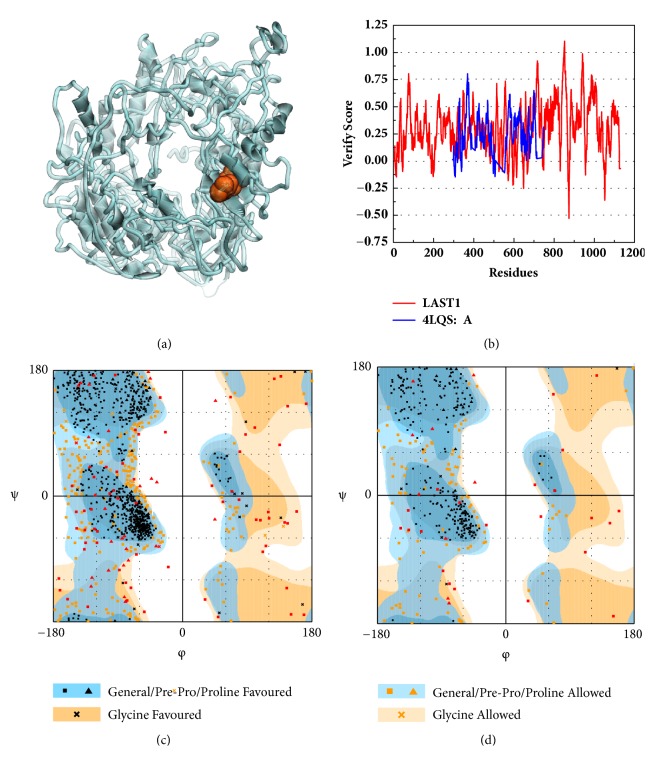
The modeling structure of LATS1 and validation analysis. (a) The modeling structure of LATS1, and the red square replaces a defined binding site. (b) The 3D-profile validation of modeling structure. The Verify Score, higher than 0, means the trusted simulation of amino acids. (c) Ramachandran plot validation of our modeling structure. The area was divided into three parts; the points stayed at the favoured and allowed implying the ration of modeling structure. (d) All the plots mean the amino acids of binding sites, to find if these important amino acids stood in best or allowed fold region.

The modeling structure of LATS1 was further verified by 3D-profile program and Ramachandran plot validation (Figure 3). The 3D-profile verify scores of the most of amino acids were higher than 0, especially the binding site region, which indicates the rationality of the modeling structure. The Ramachandran plot analysis showed whether each amino acid was in a reasonable angle, especially the amino acids of binding site displayed alone (Figure 3(d)).

### 3.3. QSAR Predicted Model

Appropriate descriptors were selected using the Genetic Function Approximation (GFA) algorithm (Table 1). And the descriptors would further be applied to create 2D QSAR model with support vector machines (SVM) and multiple linear regression (MLR) algorithm. The MLR model is built as follows.

Protein CK2A2:

(1) pIC50=6.8787−0.3456ES_Count_ssCH2+1.3063Num_Rings5−3.0854CIC−2.3988JX−0.0377Jurs_PNSA_3−0.0404Strain_Energy−0.0033PMI_X+0.7681Shadow_Ylength

Protein mtHSP70:

(2) pEC50=22.6316+0.1290ES_Count_dsCH−2.1925ES_Sum_Do+0.1225SC_3_P+0.1095Jurs_PNSA_3−0.0021PMI_X+0.4466Shadow_Ylength

Protein STK3:

(3) Predicted  Dock  score=144.47−158.52JursFPSA3+3.35JursPPSA3−156.98Jurs  RASA+0.06JursSASA+0.47JursTASA+0.14JursWNSA1−0.22JursWPSA1−1.80JursWPSA3−33.68RadOfGyration−1.21ShadowYZ

Protein LATS1:

(4) PredictedDock_score=48.25−6.84ES_Count_ssO−0.06Num_AromaticBonds+0.56Num_AtomClasses−0.85Num_ExplicitBonds−374.36Molecular_FractionalPolarSASA+430.75Molecular_FractionalPolarSurfaceArea+5.44CHI_V_1−0.79SC_3_P+0.22Jurs_SASA−25.41RadOfGyration

**Figure 4:**
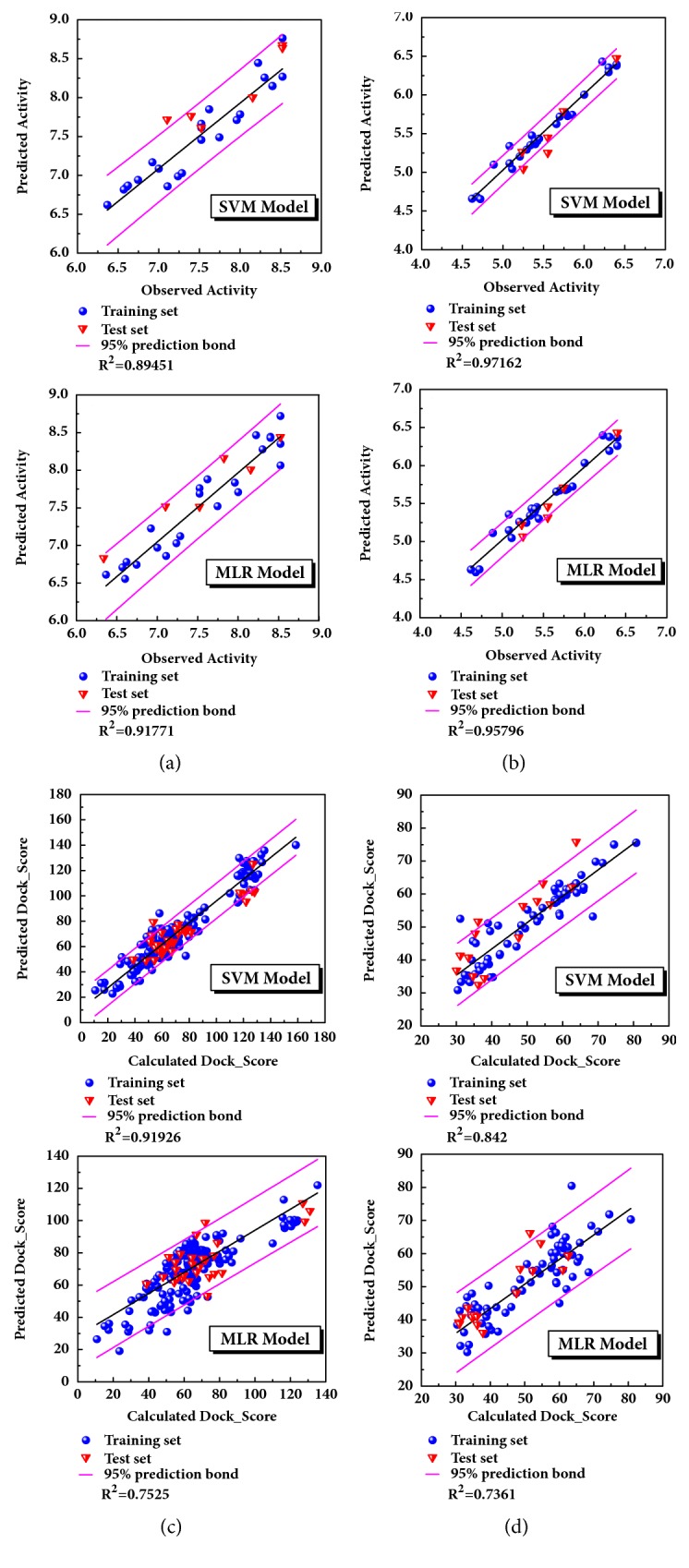
SVM predicted models and MLR predicted models for four proteins. The models constructed by the known compounds or the known formula against the targets or the disease. Protein: (a) CK2A2; (b) mtHSP70; (c) STK3; (d) LATS1.

The model was regressed by the compounds from treatment drugs. Blue spheres represented the training set, and the red inverted triangle indicated the test set (Figure 4). External verification and R^2^ were reasonable.

### 3.4. 3D-QSAR Analysis

**Figure 5:**
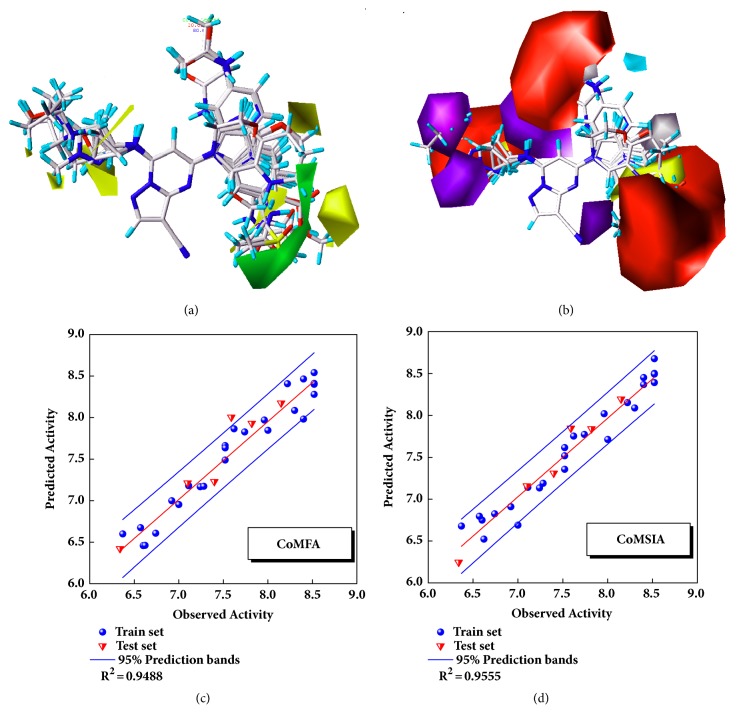
3D-QSAR model constructed by CK2A2 ligands. (a) CoMFA model; (b) CoMSIA model; (c) CoMFA model validation; (d) CoMSIA model validation.

The CoMFA and CoMSIA models of 3D-QSAR were constructed (Figure 5). The value of CoMFA and CoMSIA was calculated further to obtain the predicted activity. And the residuals between observed IC50 and predicted IC50 would be one of the evaluation values of predictive ability (Table 2). Partial least squares (PLS) analysis and validation of CoMFA and CoMSIA model provided the value of correlation coefficient (R^2^), standard error of estimate (SEE), and F test (F ratio) for evaluating, as well as the cross validation (q^2^_cv_) for assessing (Table 3). Large enough R^2^ and relatively small SEE could explain the rationality of the model. And q^2^_cv_ greater than 0.5 were worth being considered. The information of several 3D-QSAR model CoMFA analysis reminded us where bulky substituent groups were needed (green area) and where no substituent groups were demand (yellow area) (Figure 5(a)). The CoMSIA model suggested what the hydrophobic groups (yellow area), hydrophilic groups (gray area), hydrogen bonds donors (cyan area), and hydrogen bonds acceptors (violet area) needed. Areas that were inappropriate for introducing hydrogen bond acceptors would be warned correspondingly (red area) (Figure 5(b)). External validations of the models were provided to identify its accuracy (Figures 5(c) and 5(d))

### 3.5. Pharmacophore Analysis and Cross-Validation

**Figure 6:**
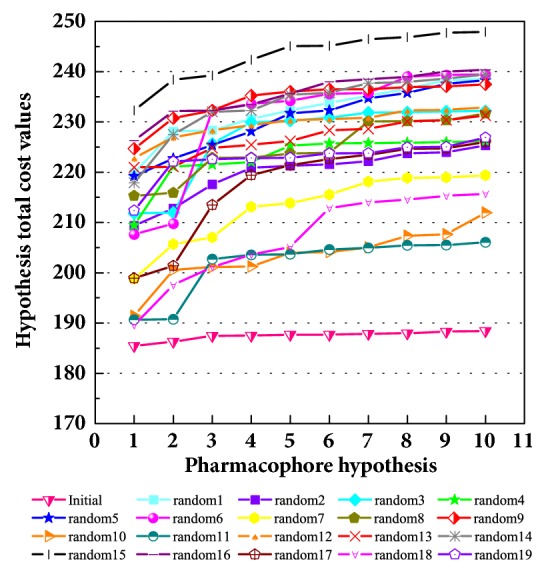
Cat-Scramble validation. The total cost of the initial spreadsheet and 19 random spreadsheets was displayed. The total cost values of all random result were higher than initial, which indicated the reliability of the pharmacophore model.

**Figure 7:**
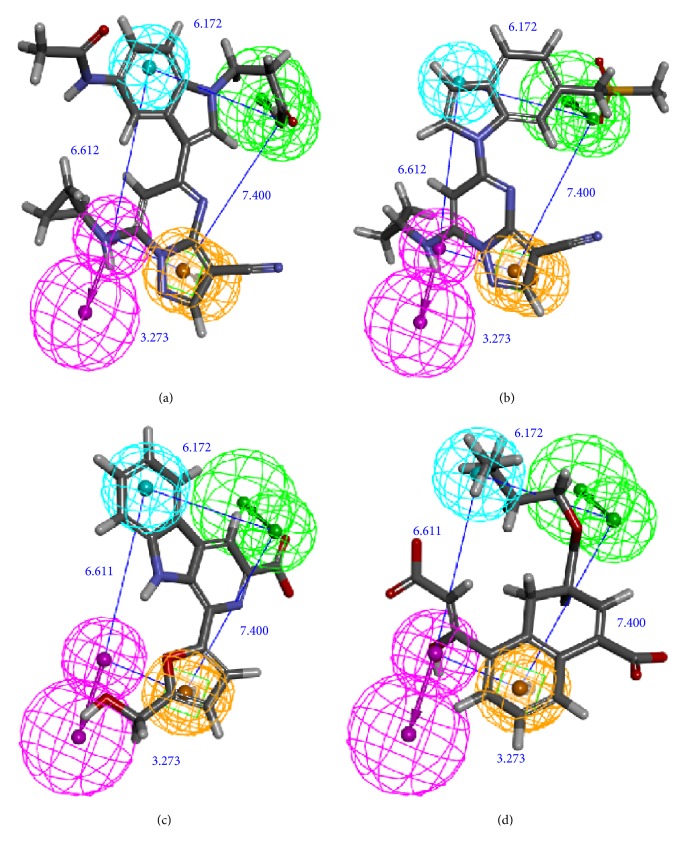
HypoGen model matched with the compounds. H-bond acceptor (green sphere), H-bond donor (pink sphere), hydrophobic (light blue sphere), and pi-interaction (orange sphere) could test and design. The distance (Å) between different pharmacophore spheres suggested the spatial distribution. (a) The match of the known best compound and pharmacophore; (b) test set molecule; (c) Candidate 1a; (d) Candidate 1b.

The activity predicts model would be verified with cross-validation. The leave one out cross-validation (LOOCV) was used to illustrate the reliability of the model. The information of 10 hypoGen models was shared (Table 4). Cat-Scramble suggested the accuracy of pharmacophore model. The total cost values of 19 random hypotheses were all higher than the initial spreadsheet (Figure 6). The difference between null cost and total cost was vital to assess the confidence of the model. Configuration cost value was 15.59 lower than 17 which at a reasonable interval. Pharmacophore Analysis further evaluated the candidates; compounds 1a and 1b nearly matched all spatial factors (Figure 7).

### 3.6. Dock Screening Result and Candidate Determination

**Figure 8:**
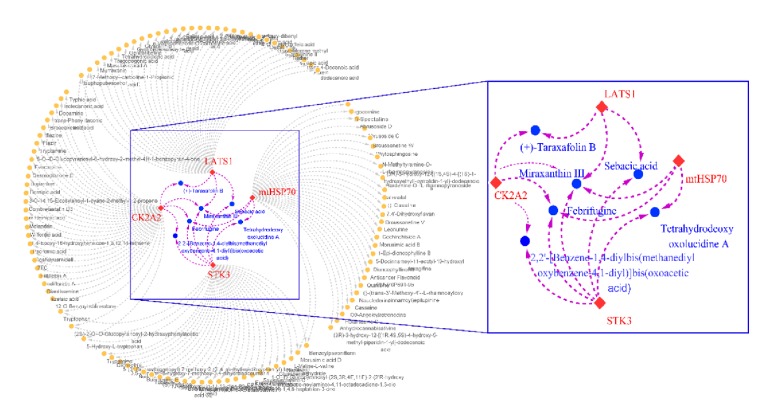
Screening result of four proteins in network pharmacology-based analysis (red diamond replaced the four targets; blue square means the selected compounds).

Top 50 compounds in each target were created in a network to suggest the multitarget effect (Figure 8). The screening rules the vote score based on the docking scores, SVM and MLR predicted activity, and consensus scores (calculated from 11 scores). For example, top 20 percent of each project was set as efficient and scored 1 point, otherwise 0 point. The total score was set as final evaluation (Table 5). The screening result for CK2A2 and mtHSP70 was displayed in Table 6, while the STK3 and LATS1 were shown in Table 7.

**Figure 9:**
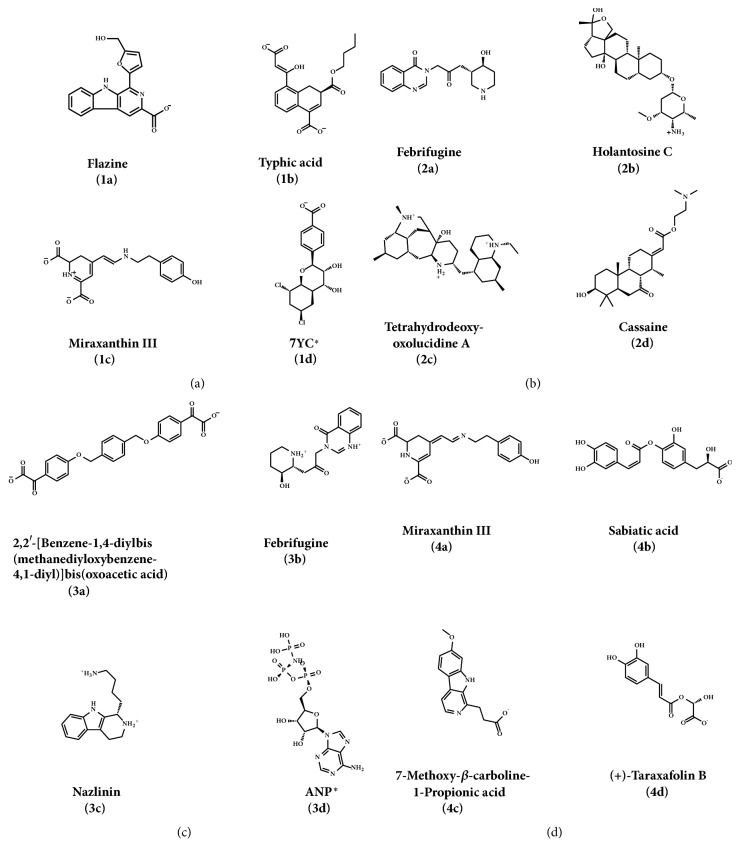
The screening result in TCM database of four related proteins. After the analysis of a scoring system with consensus score, SVM algorithm to activity predicted, and MLR algorithm to activity predicted, each related protein selects four compounds (contain control molecular). And the compounds code name is shown in brackets. Candidate: (a) CK2A2 ligands; (b) mtHSP70 ligands; (c) STK3 ligands; (d) LATS1 ligands. *∗*Control.

**Figure 10:**
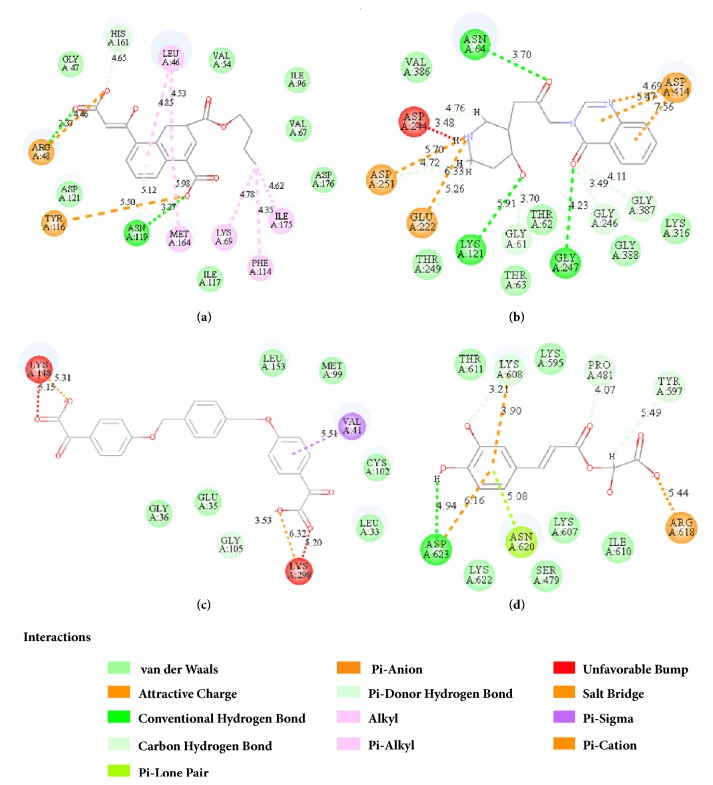
2D diagram of combined pattern in four targets complexes. The binding force included H-bond, pi-interaction, and van der Waals' force provided, and the space conflicts (red mark) were reminded. (a) CK2A2-1a complex; (b) mtHSP70-2a complex; (c) STK3-3a complex; (d) LATS1-4d complex.

2D structure of candidate compounds was displayed in Figure 9. The hydrogen bonds, hydrophobic bonds, etc. were displayed to suggest potential poses of ligands and receptors (Table 8); especially several residues binding with all ligands would be focused on. The hydrogen bonding was a significant reference for these binding analyses of combining ability. 2D diagram for different targets was shown in the interaction bonds between key residues and ligands. Van der Waals force, hydrogen bonds, salt bridge, attractive charges, pi-interaction, etc. displayed the binding potentials (Figure 10). The bond length (Å) was considered as the index of stability. Unfavorable bumps prompted space conflict that is not recommended for introduction (red callout). Probably it was a result that the ligand 3a-STK3 was not stable during MD period.

### 3.7. MD Analysis

MD results were analyzed in the Gromacs5.0.4 program.

**Figure 11:**
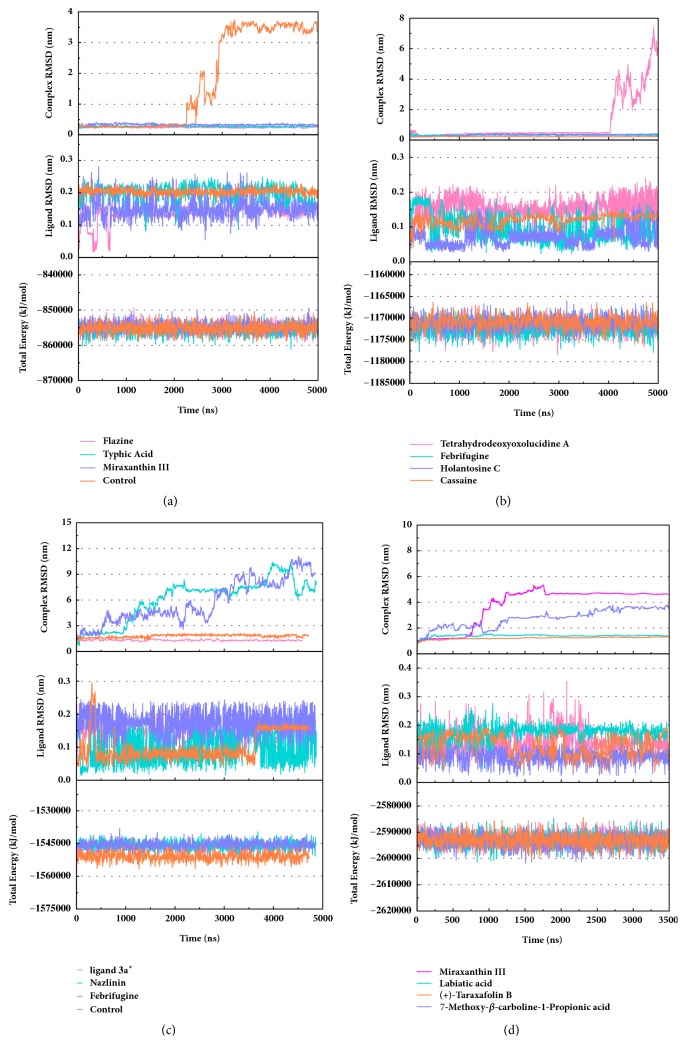
Total energy and RMSD changes during MD trajectories. (a) CK2A2 protein with three candidates and a control; (b) mtHSP70 protein with candidates; (c) STK3 protein with candidates; (d) LATS1 protein with candidates. Different colors represent different molecular candidates, which could demonstratively reveal the state of complex.

Trajectory files obtained after MD were made as videos (supported video set (available here)) for observing and displaying. They were convenient for our research by improving intuitive information. Total energy and RMSD changes (include proteins and ligands) were provided to analyze whether the receptor-ligand interaction in a proper state (Figure 11). During the MD period, the CK2A2-Flazine (1a), CK2A2-Typhic Acid (1b), mtHSP70-Febrifugine (2a), mtHSP70-Holantosine C (2b), mtHSP70-Cassaine (2d), STK3-3a, STK3-ANP (Control), and LATS1- (+)-Taraxafolin B (4d) were tested whether interactions well for a long time. Most of the complex possessed nonfluctuating total energy, even if the protein interacted with different ligands. Total energy of CK2A2 protein-ligand complex stand at nearly -860000~-850000 kJ/mol, while mtHSP70 was -1175000~-1170000kJ/mol, STK3 was -1550000~-1545000 kJ/mol, and LATS1 was -2600000~-2590000kJ/mol. It was noteworthy that the energy of STK3-control was lower compared to other ligands, which indicates the control complex stayed at a more stable state. Changes in RMSD suggested that the interactions changed in configuration. It was more concerned whether the RMSD is stable at the end period of MD. Ligand RMSD of STK3-control and LATS1-4d fluctuated obviously during MD; nevertheless, both of them did not infect the total energy or protein RMSD; the former probably changed posture at 3500 ns (Figure 11).

**Figure 12:**
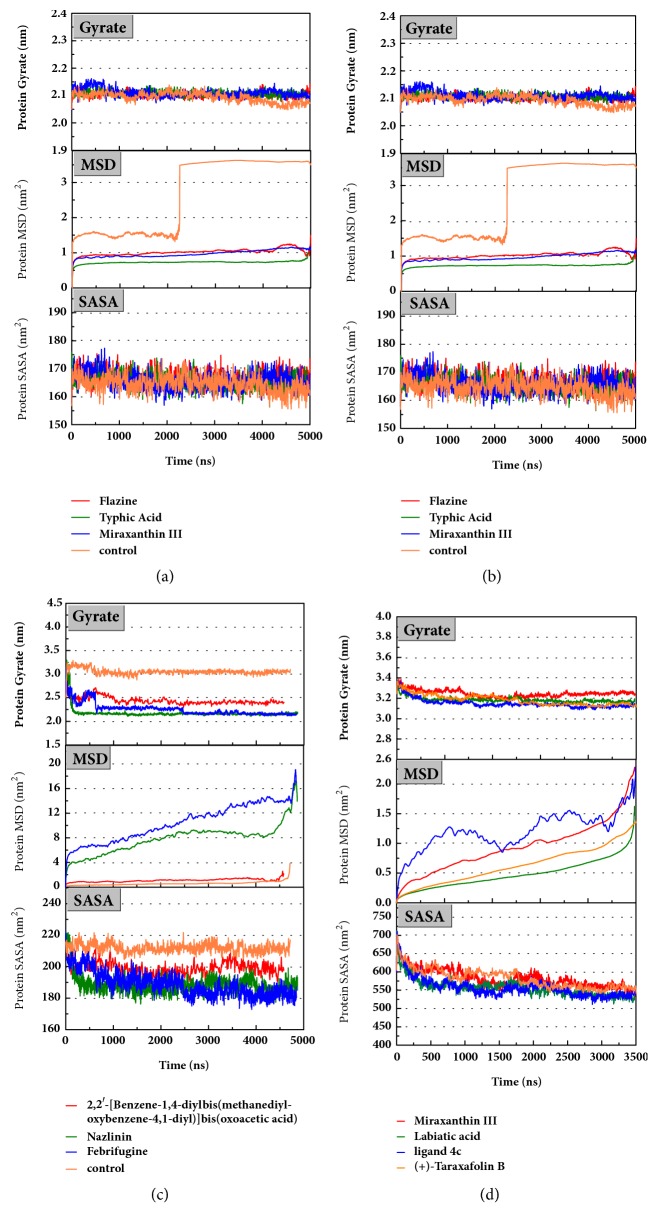
Protein gyrate, MSD, and SASA result of four targets complex with candidates. Different colors lines mean different ligand-receptor interaction. (a) CK2A2 protein; (b) mtHSP70 protein; (c) STK3 protein; (d) LATS1 protein.

**Figure 13:**
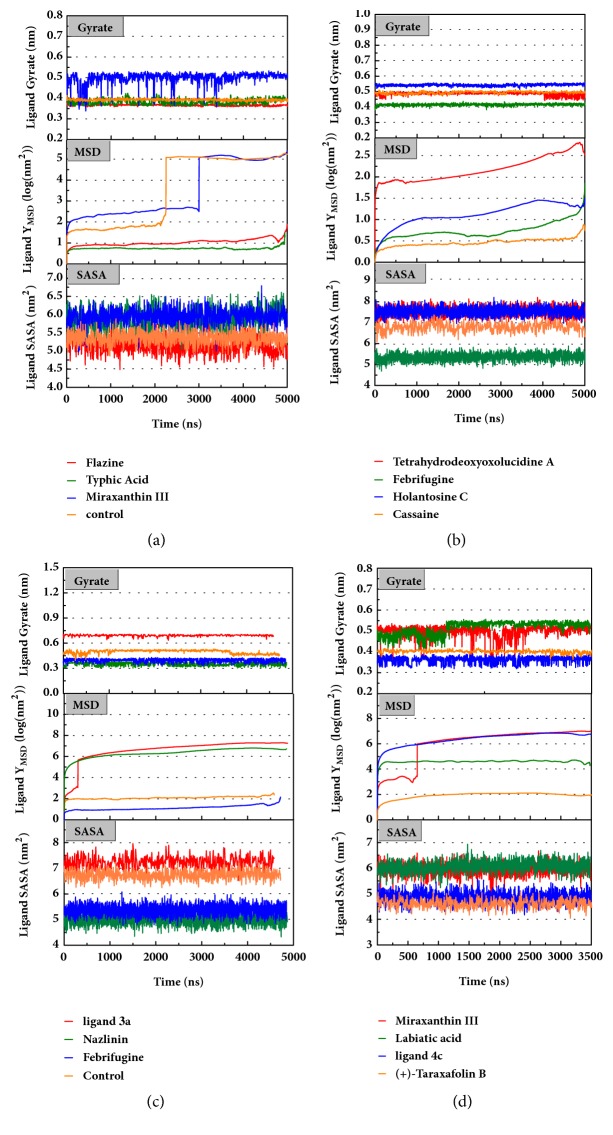
The changes of ligand gyrate, MSD, and SASA during MD simulation trajectory. Some of the ligand names were shown in Figure 9. Receptors: (a) CK2A2; (b) mtHSP70; (c) STK3; (d) LATS1.

The mean square displacement (MSD), solvent accessible surface area (SASA), and Radius of Gyration (gyrate) analysis provided the information about both protein and ligand during MD (Figures 12 and 13). Gyrate shows the more bulky the molecule, the smaller the value and the closer the molecules. In general, gyrate presents a downward trend. And for the STK3 protein STK3-control and STK-3a (red line) all displayed high gyrate, which indicate that the ligand induced fit to the protein and the protein maintain a bigger open binding site (Figure 12(c)). For mtHSP70 protein (Figure 12(b)), the opposite is true; when the protein was in a bulky state, the ligands easily “fly away”; when the protein was tighter, the ligand could get better stay inside. MSD analysis told us the protein change between the original state and simulation state. It was clearly which ligand “fly away”. The value of MSD was logarithmic transformation: Y_MSD_=2+log_10_ (MSD). In fact, several proteins remain changing at the end of simulation, even if we simulate very long time. SASA analysis could help us understand the hydrophobic nature and protein surface state. SASA of CK2A2 was little difference when combined with different ligands as well as LATS1 protein (Figures 12(a) and 12(d)). SASA of STK3 protein (Figure 12(c)) displayed higher hydrophilic when complex with ligand (red line and orange line); it was related to its bulky structure predictably.

Mean square displacement (MSD), solvent accessible surface area (SASA), and Radius of Gyration (gyrate) analysis of each ligand showed in Figure 13. The ligands' staying with protein complexes during MD would be focused. Ligand gyrate was stable in general. The ligand MSD revealed the ligand change during MD; as for STK3 protein, the ligand 3a (red line) increased at 4000 ns; it was consistent with gyrate drop, which indicates structure contraction at that time. SASA of ligands change little at MD period. One interesting condition is for STK3 protein (Figure 13(c)), where the SASA value of the ligands complex with protein (red line and orange line) was higher than other “fly away” ligands (blue line and green line); probably the bulk protein structure provides more chance for inside ligands to contact with water.

Nonbond relationship provided the binding information between the ligands and receptors; it was an important reference for drug design (Table 8). For example, it told us the Residue R48 of CK2A2 kept the H-bond, electrostatic interaction, and hydrophobic interaction. As the same, E222 of mtHSP70, D164 of STK3, and R618 of LATS1 can be significant for maintaining interaction effect.

**Figure 14:**
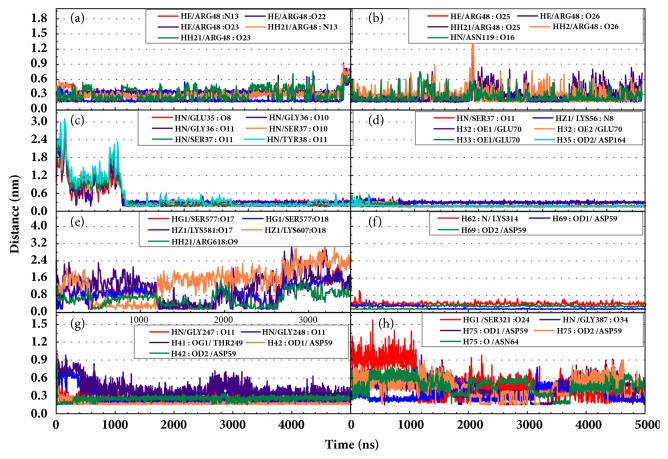
The change of hydrogen bond distance during MD period. Low distances and fairly stable hydrogen bonds were considered to be fairly favorable for the interactions. The protein-ligand interaction is replaced as (a) CK2A2-1a; (b) CK2A2-1b; (c) STK3-3a; (d) STK3-3d; (e) LATS1-4d; (f) mtHSP70-2a; (g) mtHSP70-2b; (h) mtHSP70-2d.

Hydrogen bond distance analysis was vital to explain the interaction between targets and proteins. The variation of main H-bond was observed to interpret the stability of receptor-ligand complexes (Figure 14). And the H-bond distance and the occupancy were showed to explain whether these H-bonds were in a proper distances (Table 9). The H-bond occupancy of STK3-control and mtHSP70-Febrifugine was kept high which can predict a good interaction for these complexes. As for LATS1 protein, unfortunately low H-bond occupancy indicated the ligand may leave even if we have not yet observed (Figure 14(e)). The H-bond information informed the significant residues. ARG48 and ASN119 of CK2A2 were great for maintaining complex interactions. ASP59, Gly247, Gly248, and Gly387 of mtHSP70 protein mainly built hydrogen bonds. As for STK3 protein, different ligands bound with different residues of protein (Figures 14(c) and 14(d)), the different interaction mode can be validated in further poses analysis. The observations for LATS1-4d were worrying, which had no stable hydrogen bond.

**Figure 15:**
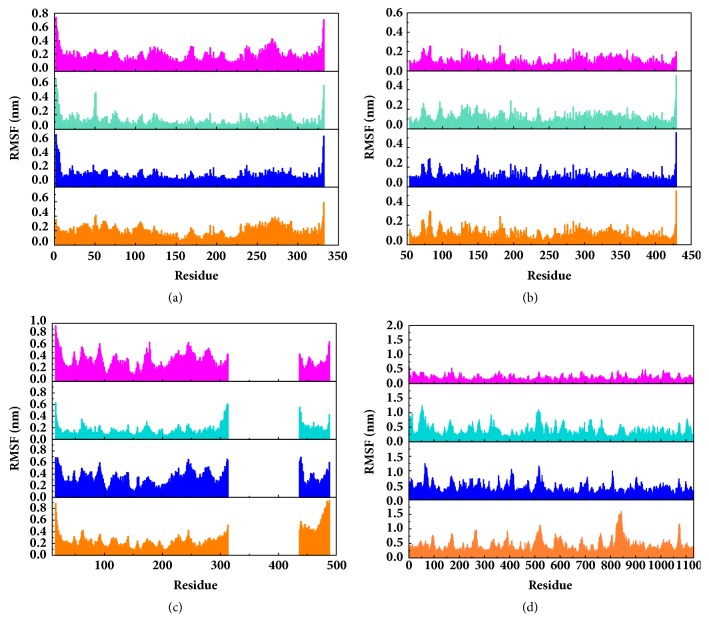
RMSF value of each residue on various proteins at the last 500 ns simulation. The abscissa was the sequence number of protein residues. This figure could visually reveal the vibration amplitude of each residue and displayed which residue has a larger range of variation. It could also compare whether the different ligands had similar effects with the targets. Protein: (a) CK2A2; (b) mtHSP70; (c) STK3; (d) LATS1.

**Figure 16:**
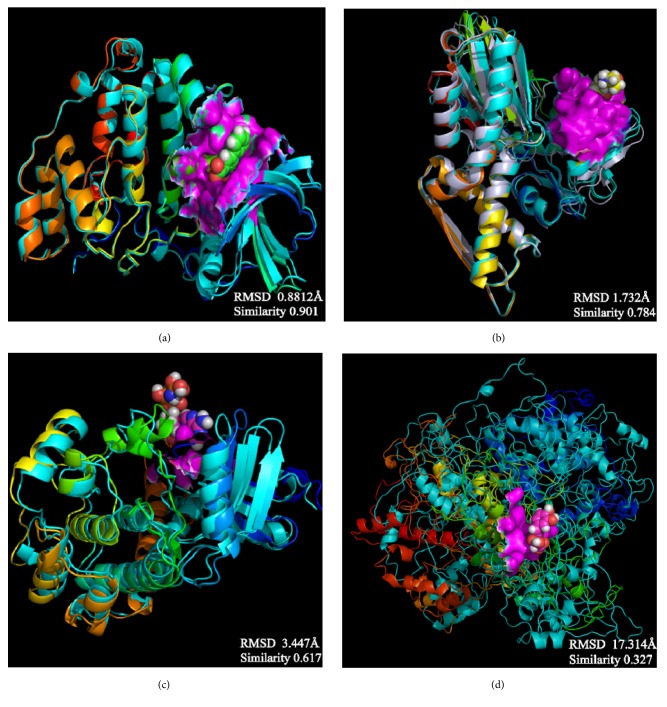
Average structure of each proteins reacted with different ligands. The average structure of the corresponding proteins was superimposed after interacting with different ligands in the same image, and these structures were to observe whether their conformational changes were consistent. The last one was the superposition of the protein that bound with ligand and the protein that the ligand had taken off during MD. It could be seen that it was completely different from the first three pictures, the protein of the figure has no similar conformation. Protein (a) CK2A2; (b) mtHSP70; (c) STK3; (d) LATS1.

Root mean square fluctuation (RMSF) can be used to test fluctuation of each residue, which can notify the key residues change during MD. The key residues contributed nonbond interaction which would mainly be focused on. CK2A2 complexes had similar residue fluctuations (Figure 15(a)), which correspondingly showed similar average protein structure (Figure 16). Residues fluctuation of mtHSP70 complexes displayed similar trend, which have the same fluctuation peaks and valleys. The difference between these complexes was the different degrees of change (Figure 15(b)). The only one ligand “fly away” (pink line) had different types of fluctuations, which further verified the role of ligands. As for STK3 protein (Figure 15(c)), ligand 3a (pink line) and control (orange line) displayed different changes mainly due to the loop area outside, which showed in different average structure (Figure 16). The change of LATS1 complexes was more complex due to a lot of residues, but it could be clearly that the fluctuation of key residues like P481, Y597, K608, and R618 influenced the binding interaction.

The average protein structures would be superimposed to observe whether the binding structures are alike, which can predict these structures related to their inhibition. CK2A2 and mtHSP70 were nearly superimposed on one structure, respectively. STK3 can be superimposed except the outside loop. The two LATS1 protein structures cannot merge into one because one ligand of structure “flies away”. It manifested the structure which acting with ligand was completely different from the origin protein (Figure 16).

The protein residues contact map provided 3D information about the residues distance of proteins in 2D matrix. Average amino acid distance was shown in different color, which indicates the looseness of protein. The distance between key residues was focused on. It demonstrated a tight conformation for SK2A2 protein. The key amino acids R48 and N119 of CK2A2 are all close to other residue that can construct a “hotspot” cavity. Similarly, key residues D59, G247, etc. of mtHSP70 constitute an active cavity site. The amino acids S37, K56, E70, and D164 of STK3 are also in a reasonable range. Different ligands for CK2A2 or mtHSP70 protein would get similar 3D structure shown in residues distance matrix, respectively, which further validate our inference above.

**Figure 17:**
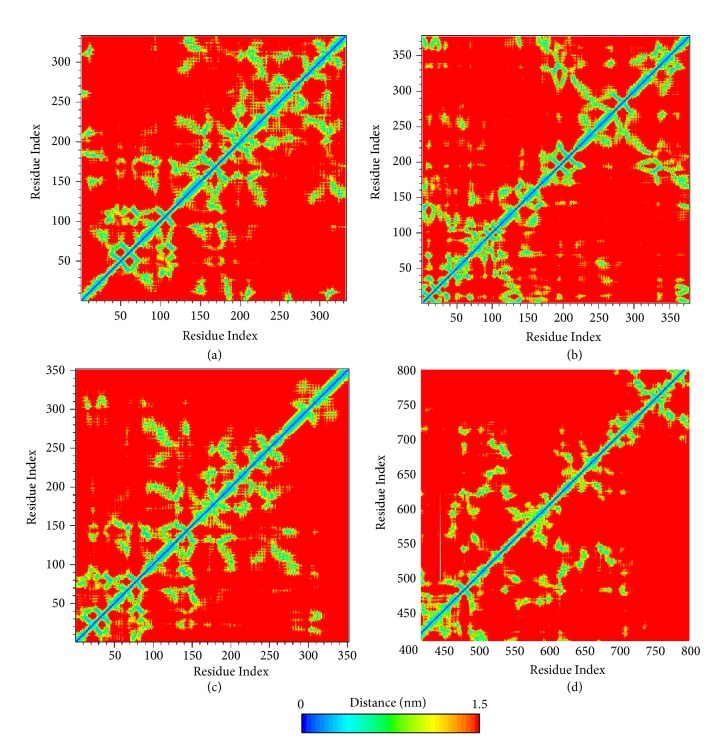
Residue distance matrix can show the distance between each residue. The main observation is the yellow-green region. It can be seen which residues were closed in space, and the residues around the binding site would be particularly concerned by us. The first three maps were mainly for all residues of the corresponding docking proteins, while the last one specifically extracts the residue information from the key region. Protein (a) CK2A2; (b) mtHSP70; (c) STK3; (d) LATS1.

Secondary structure of protein provided important information on structural stability. Maintaining A-helix and *β*-sheet during MD period can keep structural stability. Basically secondary structure was stable, including the key residues. A change was observed like residue 176-181 of mtHSP70-Febrifugine since 3000 ns, where A-helix changed to *β*-turn. It is possible that complex structure changes to a more stable configuration since this moment influenced by the ligand (Figure 17).

**Figure 18:**
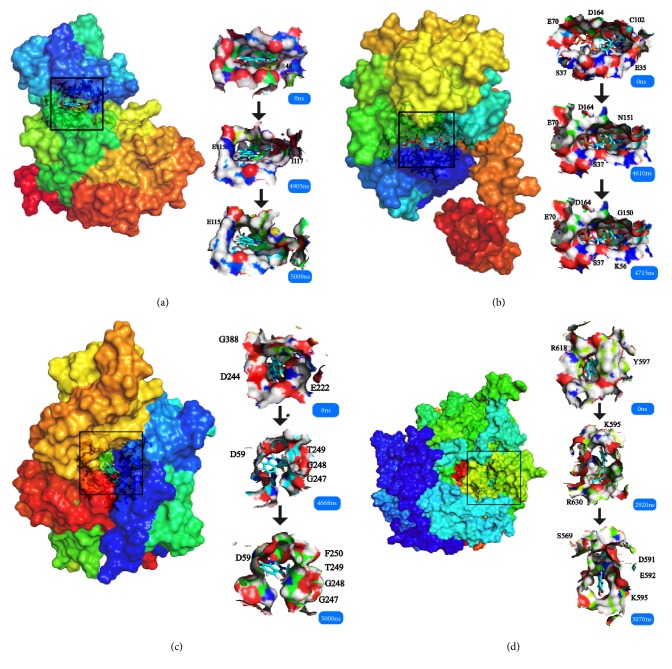
Combining posture changes during MD in microenvironment. Each complex displayed three poses, the docking poses, clustering poses among MD last 500 ns, and the ending poses. The key residues were demonstrated. The modes of last two time points were similar, but not the same as the docking position, which indicated the necessity of MD even the long-time MD. Complex: (a) CK2A2-1a; (b) STK3-3d; (c) mtHSP70-1b; (d) LATS1-4d.

Cluster analysis was taken for each complex at the last period. The dock poses, cluster poses during the last 500 ns period, and the last poses were provided (Figure 18). Cluster poses were got from the big class of cluster map. The poses between dock and the last were changed in some complex like LATS-ligand; in the large cavity of the action site, the ligand is completely steered, showing another way of acting. The CK2A2 and mtHSP70 were relatively fixed. And the H-bond relationship was shown which could clearly know the interaction modes of key residues like Asp59, Glu222, Asp 244 in the mtHSP70, etc. For the same receptor, different ligand employed different residual contributions; however, the common force mode between different complexes could help us find the key residues like Asp48 and Asn119 in the CK2A2 protein.

**Figure 19:**
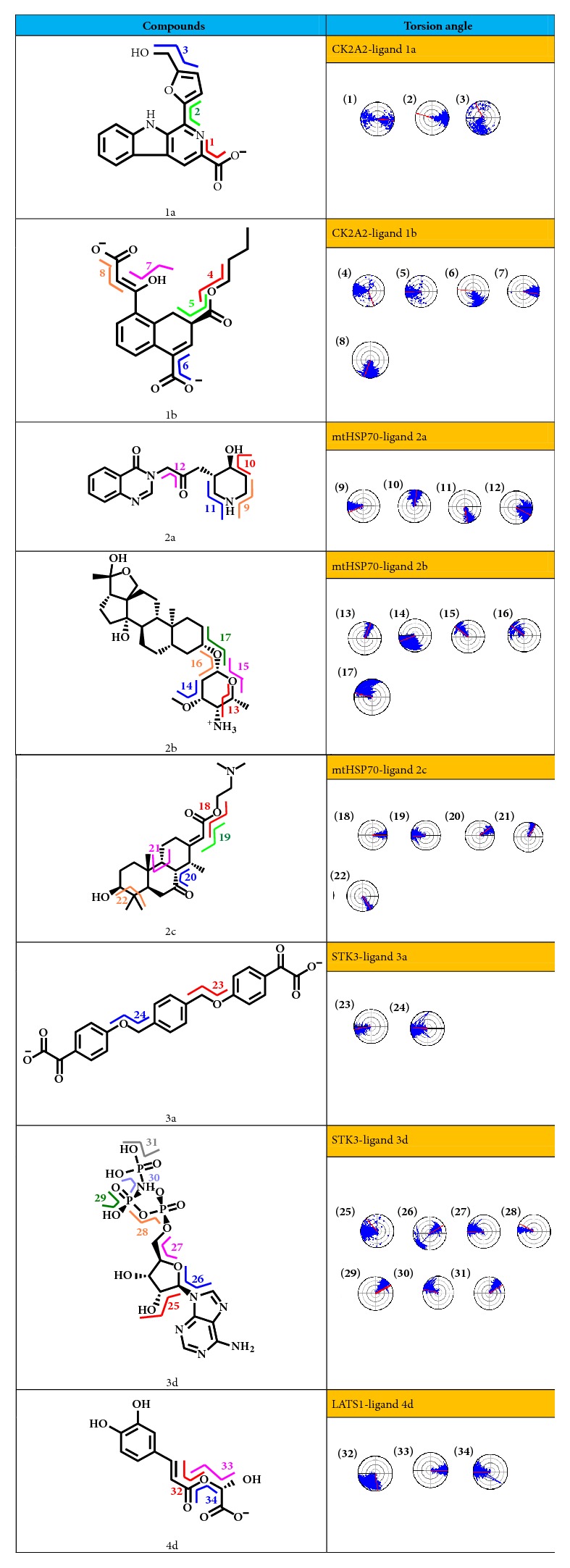
Torsion changes during MD simulation. Some single bonds that involve space or hydrogen bonds had been specifically selected for study, and it was possible to guess which hydrogen bonds can exist more stably and which structures will be bound in space to achieve a more stable combination. A single bond with a small twist angle will be noticed.

The change in the torsion angle provides important clues to the stability of hydrogen bonds. If a single key that can be rotated originally is fixed in a relatively small range of variation, it may be fixed by space or electrically fixed. Relatively speaking, finding its related hydrogen bonds also shows that the hydrogen bonds are relatively stable (Figure 19). Torsion 1 showed 180 degrees of twist, but in fact two O elements on the carboxyl were equivalent when carboxyl dissociates; hydrogen bonds which could form after 180-degree rotation were still equivalent to origin hydrogen bond; what is certain was that this was a weak hydrogen bond. Torsion 2 had a great influence on the overall conformation of the ligand molecule connecting two planes, which showed rotation of furan ring plane; its orientation can bring hydrogen bonding or hydrophobic interaction; approximately 30 degrees of change is relatively stable to conform a H-bond or hydrophobic interaction. Torsions 4-8 are all related to H-bond. The torsion angle here remained a 20-45-degree rotation; there may be hydrogen bond formation. Torsion 4 may be limited by spatial orientation as a tetra-alkyl end. Less than 20 degrees of twist in torsions 9 and 11 indicate the N can conform H-bonds; it was due to the limited effect of the ring, although it is not an aromatic ring. Torsion 10 can show the potential effect of hydroxyl. The change of torsion 12 was about 60 degrees. Mainly studying single bonds near O or N, torsions 13 and 15 are very stable with about just 10- or 20-degree rotation. Oxygenated areas around single rings were likely to form several binding forces. Torsions 18-22 rotated in a small range. The tricyclic and double bond limited made several bonds stable that may be advantageous. Most single bonds of molecular 3a rotated freely except the two single bonds 23 and 24. This may suggest that compound 3a spatially interacts with the receptor in a different way during simulation trajectory. Torsions 25-31 showed the changes of compound 3d, which further illustrated the good interaction between STK3-Control complexes. So the strategy for STK3 target was to de novo modify after screening referring to the control compound structure. O element around torsions 33-34 has the possibility of hydrogen bonding, but the hydroxy group on the phenyl ring failed to form a fixed hydrogen bond. The span of torsion 32 was nearly 90 degrees that this single would keep moving obviously.

**Figure 20:**
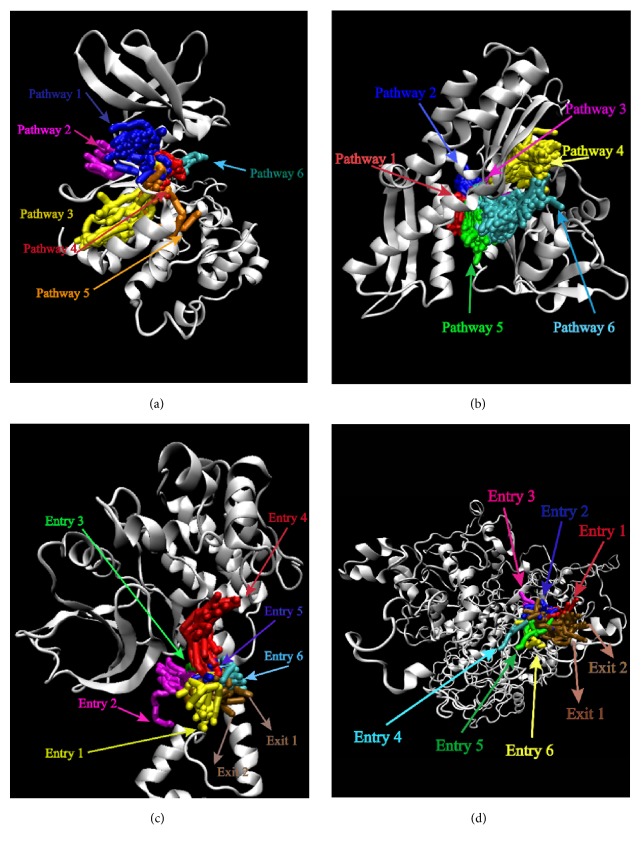
Pathway analysis for each target protein. Each color represents the path of the ligand of the protein binding site, and the latter two proteins specifically indicate the various possibilities of entry and exit. Protein: (a) CK2A2; (b) mtHSP70; (c) STK3; (d) LATS1.

Pathway analysis could provide various pathways that the ligands could entry using Caver 3.0 [40]. The possibility of ligands entering the binding region was diverse. More entry paths mean greater possibilities for integration of ligands and proteins. Almost all the targets provide lots of access routes for ligands, which is advantageous for binding (Figure 20).

## 4. Discussions

Traditional Chinese medicine provides another treatment idea with no solution strategy. Compound information of TCM was provided in our TCM database (the world's largest database of traditional Chinese medicine). Considering the docking scores comprehensively, SVM prediction activity and MLR prediction activity could explain the effect well. The verification of the simulation was provided in the experiment, such as the validation of the homology modeling and the activity prediction module.

Traditional Chinese medicine combined with network pharmacological analysis could get more effective information. It could consider multiple targets synthetically, which is quite beneficial for treatment, especially for stubborn diseases. It is impossible to employ only one target or one drug against disease nowadays. Considering the comprehensive consideration of the disease, it is exactly the same as the concept of traditional Chinese medicine. Small molecules in traditional Chinese medicine formula can be linked to multiple targets through a network, just as western medicine has been studied. Traditional Chinese medicine modernization, or integration of TCM and western medicine (WM). Network analysis can find the intercommunication between WM and TCM, bridge the current gap, and promote integrated treatment [41]. Network pharmacology-based approach integrates information into disease networks and pharmacological networks. With computational methodology, western medicine (WM) and TCM adopt networks analysis as the standard for evaluation in disease and pharmacology.

Long-term MD simulation would tell us more reliable information. In addition, the short-term MD is actually debatable; however, it was often performed at past several years. From this experiment, we want to tell the scholars who use MD to be careful about molecular dynamics simulations, especially the short-term MD. The MD simulation used in our experiment ran for 5000 ns (part of the data ran for 3000 ns due to computational resources), which is quite long for the current computing resource (2018). It could be found that some complexes that can be stably combined in tens or hundreds of nanoseconds may “fly away” in subsequent simulations. This shows that short-term MD is likely to have false positive results. Of course, our MD is not long enough but using the computational resources that can now be achieved. We want to tell the researcher there are some problems in MD, so be aware of MD.

## 5. Conclusions

Dock screening result is validated by QSAR model and molecular dynasty simulation; we got the more reliable candidate of small molecule compounds from TCM database.

Several molecular dynamics were connected to the targets through TCM formula candidates. The novel formula can achieve anti-HD effects through the multicomponent and multitarget strategy. Prescriptions and details of their small molecule effects were provided; they could form a drug-multitargets and multidrug synergistic effect against HD (Table 10). It was provided the novel TCM formula drugs with compounds in it, as well as the reacted target proteins. The novel TCM prescription proposes could be a developing method, not only for Huntington's disease, but also for other chronic illnesses. The network concept deal with disease and our method of developing Chinese medicine could be a direction for development.

## Figures and Tables

**Table 1:** Genetic Function Approximation (GFA) studies with CK2A2 protein.

**Model**	**r** ^**2**^	**r** ^**2**^ ** (adj)**	**r** ^**2**^ ** (pred)**	**RMS Residual Error**	**Friedman L.O.F.**	**S.O.R. F-value**	**S.O.R. p-value**
pIC50_1 = 7.7375 − 0.37907 *∗* ES_Count_ssCH2 + 1.241 *∗* Num_Rings5 − 3.1053 *∗* CIC − 2.7227 *∗* JX − 0.045277 *∗* Jurs_PNSA_3 − 0.04508 *∗* Strain_Energy − 0.0033914 *∗* PMI_X + 0.77384 *∗* Shadow_Ylength	0.8752	0.8299	0.7351	0.2877	0.4128	19.29	2.886e-008

pIC50_2 = -2.9109 − 0.36552 *∗* ES_Count_ssCH2 + 1.3155 *∗* Num_Rings5 − 2.7949 *∗* JX + 12.751 *∗* SIC − 0.064703 *∗* Jurs_PNSA_3 + 0.0050522 *∗* Jurs_WNSA_2 − 0.047705 *∗* Strain_Energy + 0.55252 *∗* Shadow_Ylength	0.8751	0.8296	0.7426	0.2879	0.4133	19.26	2.926e-008

pIC50_3 = -3.4898 − 0.37365 *∗* ES_Count_ssCH2 + 1.3929 *∗* Num_Rings5 + 14.011 *∗* BIC − 2.4178 *∗* JX − 0.073379 *∗* Jurs_PNSA_3 + 0.006691 *∗* Jurs_WNSA_2 − 0.046048 *∗* Strain_Energy + 0.56523 *∗* Shadow_Ylength	0.8747	0.8291	0.7400	0.2884	0.4146	19.19	3.026e-008

pIC50_4 = -8.4336 − 0.37402 *∗* ES_Count_ssCH2 + 1.2103 *∗* Num_Rings5 − 2.1622 *∗* JX + 14.991 *∗* SIC − 0.044757 *∗* Jurs_PNSA_3 − 0.044286 *∗* Strain_Energy − 0.0039811 *∗* PMI_X + 0.80898 *∗* Shadow_Ylength	0.8743	0.8286	0.7325	0.2888	0.4158	19.13	3.116e-008

GFA algorithm is also constructed in other target proteins the same way.

**Table 2:** Predicted CoMFA and CoMSIA models.

Comp.	pIC50	CoMFA			CoMSIA				
		Value	Pred*∗*	Resid	D&A	S&E	Hy	Pred*∗*	Resid
1	7	80	6.953	0.047	1.97	4.11	2.49	6.691	0.309
2	6.74	84	6.609	0.131	1.97	4.38	4.01	6.825	-0.085
3	7.24	98	7.168	0.072	1.97	4.67	3.64	7.135	0.105
4	6.37	84	6.6	-0.23	1.97	4.39	4.50	6.679	-0.309
5	6.34	102	6.422	-0.082	1.75	4.73	2.62	6.247	0.093
6	7.11	118	7.18	-0.07	1.75	5.24	3.78	7.141	-0.031
7	7.28	124	7.174	0.106	1.75	5.43	4.15	7.19	0.09
8	6.6	112	6.46	0.14	1.75	5.20	2.77	6.751	-0.151
9	8	130	7.847	0.153	2.86	5.86	4.02	7.713	0.287
10	7.59	130	8.004	-0.414	2.82	5.75	3.52	7.847	-0.257
11	8.52	132	8.28	0.24	2.81	5.76	3.83	8.393	0.127
12	8.4	140	8.465	-0.065	2.63	5.92	3.67	8.369	0.031
13	6.57	144	6.675	-0.105	3.02	6.05	3.82	6.796	-0.226
14	6.62	150	6.462	0.158	3.23	6.16	3.62	6.524	0.096
15	7.1	140	7.212	-0.112	2.86	6.05	4.16	7.158	-0.058
16	7.62	134	7.865	-0.245	2.86	6.03	4.37	7.754	-0.134
17	8.4	138	7.982	0.418	3.45	5.92	4.55	8.453	-0.053
18	7.52	150	7.49	0.03	3.41	6.16	4.28	7.519	0.001
19	7.52	148	7.664	-0.144	3.67	6.26	4.99	7.358	0.162
20	7.4	164	7.229	0.171	2.89	6.56	4.97	7.31	0.09
21	7.52	164	7.636	-0.116	2.89	6.41	4.36	7.616	-0.096
22	7.74	170	7.828	-0.088	2.89	6.72	4.81	7.774	-0.034
23	7.96	146	7.971	-0.011	2.54	6.04	4.01	8.022	-0.062
24	6.92	130	6.999	-0.079	2.64	5.60	3.69	6.911	0.009
25	8.15	144	8.174	-0.024	3.32	5.98	5.26	8.195	-0.045
26	8.22	146	8.408	-0.188	2.82	6.00	3.93	8.154	0.066
27	8.3	136	8.087	0.213	2.82	5.81	3.84	8.089	0.211
28	8.52	158	8.542	-0.022	3.39	6.25	4.55	8.503	0.017
29	8.52	170	8.401	0.119	3.40	6.42	4.53	8.678	-0.158
30	7.82	170	7.931	-0.111	3.36	6.51	4.39	7.841	-0.021
31	8.52	158	8.412	0.108	3.67	6.37	4.29	8.495	0.025

*∗*pred: predicted pIC50.

Resid: residual.

S: steric.

Hy: hydrophobic.

D: hydrogen bond donor.

A: hydrogen bond acceptor.

E: electronic effect.

**Table 3:** PLS analysis and validation of several CoMFA and CoMSIA models.

Parameter	q^2^_cv_	ONC	r^2^	SEE	F ratio
CoMFA*∗*	0.536	6	0.941	0.189	64.014
CoMSIA					
S	0.469	6	0.858	0.294	24.072
H	0.513	5	0.915	0.221	53.863
D	0.525	6	0.748	0.391	11.886
A	0.477	10	0.864	0.315	12.741
S+H	0.501	5	0.925	0.209	61.907
S+D	0.545	6	0.854	0.298	23.397
S+A	0.471	6	0.906	0.239	38.434
H+D	0.557	6	0.952	0.171	79.12
H+A	0.527	5	0.913	0.225	52.684
D+A	0.509	4	0.854	0.286	38.084
S+H+D*∗*	0.564	6	0.955	0.166	84.237
S+H+A	0.505	8	0.967	0.141	118.495
S+D+A	0.529	5	0.882	0.262	37.55
H+D+A	0.521	6	0.957	0.161	89.404
S+H+D+A	0.534	6	0.957	0.162	89.264

*∗*Selected model.

q^2^_cv_: correlation coefficient (cross-validation).

r^2^: correlation coefficient (non-cross-validation).

ONC: optimal number of components.

SEE: standard error of estimate.

F ratio: F-test value.

S: steric.

H: hydrophobic.

D: hydrogen bond donor.

A: hydrogen bond acceptor.

Filed proportion (S+H+D+A): S: 10.1%; H: 36.3%; D: 21.1%; A: 32.5%.

**Table 4:** Top 10 pharmacophore models of CK2A2 inhibitors generated by HypoGen protocol.

**Hypothesis**	**Total cost**	**Correlation**	**Error**	**RMS**	**Feature** ^**b**^
Hypo 1	185.455	0.7584	168.342	1.62	HBA,HBD,HY,RING
Hypo 2	186.266	0.7513	169.514	1.64	HBA,HBD
Hypo 3	187.455	0.7463	170.365	1.655	HBA,HBD,HY
Hypo 4	187.478	0.7457	170.459	1.656	HBA,HBD,HY,RING
Hypo 5	187.622	0.743	170.899	1.663	HBA,HBD,HY
Hypo 6	187.69	0.7454	170.518	1.657	HBA,HBD,HY,RING
Hypo 7	187.872	0.7434	170.854	1.663	HBA,HBD
Hypo 8	187.973	0.7488	169.985	1.648	HBA,HBD,HY,RING
Hypo 9	188.302	0.7432	170.898	1.663	HBA,HBD,HY,RING
Hypo 10	188.414	0.7421	171.08	1.667	HBA,HBD,HY,RING

a. Cost value: Null cost = 232.267, Fix cost = 137.8, and configure cost = 15.5857.

b. Feature description: HBA, HBD, HY, and RING indicate hydrogen bond acceptor, hydrogen bond donor, hydrophobic, and Ring aromatic, respectively.

**Table 5:** Docking score of several TCM compounds with CK2A2 protein and the Voting Score of each compounds based on the related terms ranking. The selecting model will be applied to other target proteins.

Compound name	DOCK SCORE	SVM		MLR		Consensus Score		Total Score
		pIC	Vote	pIC	Vote	CS	Vote	
		50		50				
*∗*Miraxanthin III	114.346	7.08	0	8.45	1	4	1	2
*∗*flazine	96.004	7.98	1	4.63	0	4	1	2
*∗*Typhic acid	97.811	7.08	0	5.01	0	7	1	1
*∗*7FC(control)	108.017	7.06	0	6.52	1	7	1	2
2,2′-[Benzene-1,4-diylbis	96.297	7.08	0	5.94	0	7	1	1
(methanediyloxybenzene-4								
1-diyl)]bis(oxoacetic acid)								
Tryptophan	96.459	7.42	1	5.94	0	1	0	1
Nodifloridin A	109.704	7.06	0	4.78	0	6	1	1
3-O-14,15-Eicosylenoyl-1-cyano-2-methyl-1,2-propene	101.287	7.08	0	4.36	0	5	1	1
Melandrin	102.897	6.21	0	6.14	1	3	0	1
dopamine	100.157	6.93	0	6.02	1	1	0	1
Fritillebeinol	97.4	7.16	0	5.96	1	1	0	1
Tryptamine	97.061	7.33	1	4.61	0	1	0	1
Evocarpine	99.54	7.63	1	4.23	0	1	0	1
(2S)-2-O--D-Glucopyranosyl-2-hydro-xyphenylacetic acid	98.628	7.34	1	3.80	0	1	0	1
5-Hydroxy-L-tryptophan	99.271	7.49	1	3.29	0	1	0	1
8-O--D-Glucopyranosyl-6-hydroxy	98.707	7.30	1	3.20	0	1	0	1
-2-methyl-4H-1-benzopyran-4-one								
1,4-Epoxy-16-hydroxy-	104.582	6.53	0	3.82	0	3	0	0
heneicos-1,3,12,14-tetraene								
Desmodianone E	100.083	6.19	0	2.62	0	2	0	0
Combretastatin D3	101.461	6.00	0	4.13	0	1	0	0
GoshuyuamideII	107.088	6.90	0	3.81	0	1	0	0

*∗*Selected compounds after screening

**Table 6:** Candidate compounds screened out in this trial for CK2A2 and mtHSP70 protein.

Compound name	DOCK SCORE	SVM		MLR		Consensus		Total Score
		pIC	Vote	pIC	Vote	CS	Vote	
		50		50				
Miraxanthin								
III	114.346	7.08	0	8.45	1	4	1	2
Flazine	96.004	7.98	1	4.63	0	4	1	2
Typhic acid	97.811	7.08	0	5.01	0	7	1	1
7FC(control)	108.017	7.06	0	6.52	1	7	1	2
Tetrahydrodeox								
-yoxolucidine A	119.845	6.88	1	6.10	1	3	1	3
Febrifugine	107.889	6.87	1	6.93	1	3	1	3
Holantosine C	162.286	6.86	1	6.45	1	2	0	2
Cassaine	109.044	6.86	1	7.39	1	2	0	2

**Table 7:** Docking score and predicted docking score values from SVM model and MLR models of the TCM candidates.

Compound name	DOCK_SCORE	SVM predicted score	MLR predicted score	Con-sensus score
Nazlinin^a^	158.538	140.18	108.541	1
2,2′-[Benzene-1,4-diylbis(methanedi yloxybenzene-4,1-diyl)]bis(oxoacetic acid)^a^	133.038	132.538	83.646	3
Febrifugine^a^	127.99	104.625	95.46	4
ANP(control)^a^	109.987	102.144	85.874	5
Miraxanthin III^b^	71.273	69.4458	66.639	4
Labiatic acid^b^	60.03	69.8435	45.035	6
7-Methoxy-*β*-carboline-1-Propionic acid^b^	68.524	53.2091	64.378	5
(+)-Taraxafolin B^b^	65.96	62.1113	63.304	3

a. Top candidates of STK3 protein.

b. Top candidates of LATS1 protein.

**Table 8:** Receptor-ligand nonbond relationship observed in four target proteins during docking.

**Protein**	**Candidates**	**Residues**													
CK2A2		L46	G47	R48	V54	V67	K69	F114	Y116	N119	H161	M164	L175		
	Flazine	Hb	H	E;H	Hb	Hb	-	-	-	Hb	-	Hb	Hb		
	Typhic Acid	Hb	-	E;Hb;H	-	-	Hb	Hb	-	H	H	Hb	Hb		
mtHSP70		D59	G61	N64	T86	K121	E222	D244	G246	G247	D251	E313	K316	G387	D414
	Febrifugine	-	H	H	-	H	E;H	H	H	H	E;H	-	-	H	E
	Holantosine C	H	-	-	-	-	E;H	-	H	H	E	-	-	-	-
	Cassaine	-	-	-	H	-	E;H	E;H	H	H	E	H	H	-	-
STK3		G36	S37	Y38	V41	K56	E70	G105	K148	L153	D164	K298			
	2,2′-[Benzene-1,4-diylbis- (meth	-	-	-	Hb	-	-	H;E	H;E	-	E	E			
	anediyloxybenzene-4,1-diyl)]-														
	bis(oxoacetic acid)														
	Control	H	H	H	Hb	H	E	-	-	Hb	E;H	-			
LATS1		P481	Y597	K608	R618	N620	D623								
	(+)-Taraxafolin B	H	H	H;E	H;E	P	H;E								

*∗*H: H-bond, E: electrostatic interaction, and Hb: hydrophobic interaction.

**Table 9:** Main H-bond distance occupancy information for complexes.

**H-bond**	**Occupancy**	**Maximum (nm)**	**Minimum (nm)**	**H-bond**	**Occupancy**	**Maximum (nm)**	**Minimum (nm)**
CK2-Flazine				CK2-Typhic Acid			
HE/ARG48:N13	0.647	0.931	0.222	HE/ARG48:O25	0.796	1.254	0.156
HE/ARG48:O22	0.564	0.894	0.151	HE/ARG48:O26	0.838	1.437	0.150
HE/ARG48:O23	0.731	0.679	0.151	HH21/ARG48:O25	0.697	1.323	0.148
HH21/ARG48: N13	0.783	0.891	0.190	HH21/ARG48:O26	0.759	1.519	0.149
HH21/ARG48:O23	0.606	0.765	0.156	HN/ASN119:O16	0.848	0.883	0.158
STK3-2,2′-[Benzene-1,4-diylbis(methanediyl-ox ybenzene-4,1-diyl)]bis(oxoacetic acid)				STK3-Control			
HN/GLU35:O8	0.627	2.441	0.177	HN/SER37:O11	0.964	0.452	0.154
HN/GLY36:O10	0.739	2.937	0.154	HZ1/LYS56:N8	0.919	0.559	0.220
HN/GLY36:O11	0.743	2.928	0.158	H32:OE1/GLU70	0.993	0.490	0.150
HN/SER37:O10	0.752	2.962	0.156	H32:OE2/GLU70	0.994	0.403	0.145
HN/SER37:O11	0.747	2.976	0.154	H33:OE1/GLU70	0.992	0.542	0.139
HN/TYR38:O11	0.621	3.100	0.159	H35:OD2/ASP164	0.984	0.531	0.151
LATS1-(+)-Taraxafolin B				mtHSP70-Cassaine			
HG1/SER577:O17	0.181	2.211	0.155	H62: N/LYS314	0.063	1.016	0.257
HG1/SER577:O18	0.194	2.051	0.156	H69: OD1/ASP59	0.596	0.562	0.144
HZ1/LYS581:O17	0.168	3.049	0.150	H69: OD2/ASP59	0.596	0.426	0.148
HZ1/LYS607:O18	0.158	3.026	0.152				
HH21/ARG618:O9	0.241	1.473	0.167				
mtHSP70-Febrifugine				mtHSP70-Holantosine C			
HN/GLY247: O11	0.852	0.800	0.156	HG1/SER321:O24	0.313	1.569	0.167
HN/GLY248: O11	0.740	1.009	0.169	HN/GLY387:O34	0.617	0.781	0.185
H41:OG1/THR249	0.448	0.982	0.160	H75:OD1/ASP59	0.253	0.984	0.148
H42:OD1/ASP59	0.997	0.990	0.143	H75:OD2/ASP59	0.274	0.903	0.146
H42:OD2/ASP59	0.997	0.849	0.147	H75:O/ASN64	0.201	0.786	0.159

**Table 10:** The connection of potential TCM formula and HD.

Origin TCM	Compounds	Targets
Brucea javanica	flazine	CK2A2
	azelaic acid	CK2A2
	bruceine F	CK2A2
	azelaic acid	STK3
	bruceine F	STK3
Dichroa febrifuga	Febrifugine	CK2A2
	Febrifugine	mtHSP70
	A-Dichroine	STK3
	*β*-Dichroine	STK3
	Febrifugine	STK3
E. micranthum Harms	Cassaidine	mtHSP70
	Cassaine	mtHSP70
Erythrophleum guineense	Cassaidine	mtHSP70
	Cassaine	mtHSP70
Holarrhena antidysenterica	Holantosine A	mtHSP70
	Holantosine C	mtHSP70
	Holantosine D	mtHSP70
Japanese Ardisia Herb	2,2′-[Benzene-1,4-diylbis(methanediyl- oxybenzene-4,1-diyl)]bis(oxoacetic acid)	CK2A2
	2,2′-[Benzene-1,4-diylbis(methanediyl- oxybenzene-4,1-diyl)]bis(oxoacetic acid)	STK3
Taraxacum formosanum	(+)-Taraxafolin B	CK2A2
	(+)-Taraxafolin B	LATS1
Typha angustifolia	Typhic Acid	CK2A2
	Typhic Acid	STK3

## Data Availability

All data generated or analysed for this study are included in this publish article. Raw data are available from the corresponding author on reasonable request.

## Abbreviations

HD: Huntington's disease
TCM: Traditional Chinese medicine
QSAR: Quantitative structure-activity relationship
MLR: Multiple linear regression
SVM: Support vector machine
CoMFA: Comparative molecular field analysis
CoMSIA: Comparative molecular similarity index analysis
MD: Molecular dynamics
HSF1: Heat shock transcription factor 1
CK2A2: Casein kinase II subunit alpha'
Htt: Huntingtin
ER: Endoplasmic reticulum
YAP: Yes-associated protein
KEGG: Kyoto Encyclopedia of Genes and Genomes
mtHSP70: Mitochondrial 70 kDa heat shock protein
STK3: Serine/threonine-protein kinase 3
PDB: Protein data bank
DS: Discovery studio
CHARMm: Chemistry at HARvard Macromolecular Mechanics
GFA: Genetic Function Approximation
LOOCV: Leave one out cross validation
RMSD: Root-mean-square deviation
RMSF: Root mean square fluctuation
MSD: Mean square displacement
SASA: Solvent accessible surface area
gyrate: Radius of gyration.

## Supplementary Materials

Supplementary Materials All of the support videos were about receptor-ligand complex during molecule dynamics simulation periods. A candidate could bind well with protein; however, what we should care about more was stability. The MD video could intuitively reflex the stability of complexes. A compound could “fly away” in the MD period even if it presented perfect in docking. What is more, we provide these videos to display the binding modes of different complexes for support information to suggest mechanism.
